# The prevalence and associated factors of depression in policing: a cross sectional study in Sri Lanka

**DOI:** 10.1186/s40064-016-3474-9

**Published:** 2016-10-12

**Authors:** Nuwan D. Wickramasinghe, Pushpa R. Wijesinghe, Samath D. Dharmaratne, Suneth B. Agampodi

**Affiliations:** 1 Department of Community Medicine, Faculty of Medicine and Allied Sciences, Rajarata University of Sri Lanka, Anuradhapura, Sri Lanka; 2 Epidemiology Unit, Ministry of Health, No. 231, De Saram Place, Colombo 10, Sri Lanka; 3 Department of Community Medicine, Faculty of Medicine, University of Peradeniya, Peradeniya, Sri Lanka

**Keywords:** Depression, Policing, PDS, Sri Lanka

## Abstract

**Background:**

Policing is regarded as a high-risk profession for the development of mental health disturbances owing to various critical incidents and potential traumatic events they encounter. Exploration of mental health problems in policing in Sri Lanka, which recently concluded a civil war expanded over three decades, is a timely, yet, a neglected issue. Hence, the present study was conducted with the aim of determining the prevalence and associated factors of depression among police officers in the Kandy police division, Sri Lanka.

**Methods:**

A cross sectional study was conducted using a simple random sample of 750 police officers employed in the Kandy police division, Sri Lanka. A self administered questionnaire, including “Peradeniya Depression Scale” to assess depression, was used to collect data. The prevalence of depression was calculated as point prevalence with 95 % confidence intervals. Multivariable logistic regression was carried out using backward elimination method to quantify the association between depression and selected predictors identified at bivariate analysis at p < 0.10.

**Results:**

A total of 750 Police officers were invited for the study. The response rate was 94.5 % (n = 709). The mean age of the police officers in the sample was 39.6 years (SD 9.2 years). Majority of police officers (n = 591, 83.4 %) were males. The estimated prevalence of depression in the study sample was 22.8 % (95 % CI 19.9–26.1 %). However, the adjusted prevalence of depression was 10.6 % (95 % CI 6.6–15.1 %). In the multivariable analysis, of the postulated occupational factors, satisfactory welfare facilities at work place was negatively associated with depression (adjusted OR 0.5; 95 % CI 0.3–0.7; p = 0.001). Satisfaction of the opportunity to serve the public (adjusted OR 0.2; 95 % CI 0.1–0.6; p = 0.003) and satisfaction related to social status gained in policing (adjusted OR 0.5; 95 % CI 0.3–0.8; p = 0.04) were identified as significant occupational factors that lowered the likelihood of being categorized as having depression.

**Conclusions:**

The prevalence of depression among police officers was found to be higher in comparison to other study findings in Sri Lanka. Given the modifiable nature of the significant predictors, it is recommended to design a package of interventions and implement adaptive measures to rectify the problems related to depression among police officers.

## Background

Police officers play an integral part in maintaining the nation’s security, with primary focus on internal security. Their functions, apart from matters connected with the safety, comfort and convenience of the people, are connected with prevention and detection of crime and maintenance of law and order. This range of police work has been identified as an exceptionally stressful occupation (Alexander 1999; Violanti and Paton 1999; Anshel 2000; van der Velden et al. 2013). Policing deals with a psychologically stressful work environment associated with dangers and most often with human miseries. Amidst the stressful working environment police officers are often subjected to harsh media criticism and scant public gratitude (Chermak et al. 2006; Callanan and Rosenberger 2011). Thus, in such an environment, they become a highly vulnerable category of human service provision for mental health problems.

Studies across the globe reveal that depression (Chen et al. 2006; Hartley et al. 2007; Wang et al. 2010; Lawson et al. 2012; Bowler et al. 2016; Komarovskaya et al. 2014; Garbarino et al. 2013; Wang and Leather 2016), stress (Pancheri et al. 2002; Newman and Rucker-Reed 2002; Berg et al. 2006; Violanti et al. 2006; Waters and Ussery 2007), anxiety (Rukšenas et al. 2009; Evans et al. 1992), post traumatic stress syndrome (Storch and Panzarella 1996; Gersons 1989; Carlier et al. 1996; Meffert et al. 2008; Lilly et al. 2009), burnout (Kop et al. 1999; Hawkins 2001; Malach-Pines and Keinan 2006; Martinussen et al. 2007; de la Fuente Solana et al. 2013), sleep disorders (Rajaratnam et al. 2011; Ma 2011) and even suicides (Hem et al. 2001; Berg et al. 2003; Violanti 2004; Violanti et al. 2009; O’Hara et al. 2012) are common among law enforcing officers. Furthermore, studies highlight that the mental health problems among police officers are on the rise following anthropogenic and natural disasters (Duckworth 1991; Renck et al. 2002; Huizink et al. 2006; Perrin et al. 2007; Slottje et al. 2007). Rates of major depression disorder and levels of depression symptoms are reported as higher among police officers than the general population (Chen et al. 2006). The reported prevalence of depression has a wide range from 21.6 % among Taiwan police officers (Chen et al. 2006) to 65.6 % among Australian Police officers (Lawson et al. 2012). Albeit, the greater significance highlighted in many studies, there’s a scarcity in the literature pertaining to depression in policing in the South Asian region.

The concept of policing in Sri Lanka started with the establishment of police stations in the island by the Dutch. However, in 1805, the police functions were clearly defined under British ruling. Apart from matters connected with the safety, comfort and convenience of the people, police functions were defined in relation to prevention and detection of crime and maintenance of law and order. There are several specialized units and divisions in Sri Lankan police service. These specialized units and divisions include protective units, counter-terrorist units, crime-investigation units, law enforcement, support units and technology infrastructure. Provided the numerous adversaries that police officers are exposed to, a separate welfare division is established in Sri Lankan police service, in which the services have been extended to provide assistance to uplift the living standards, support to children’s education, free provision and access to medical facilities (http://www.police.lk/).

Sri Lankan law enforcing officers had to deal with many anthropogenic and natural disasters in the recent past. Amongst them, 30 years of war against terrorism, massive natural disasters like tsunami and turbulent political milieu common to the South Asian region had major impacts in policing throughout the island. A comprehensive search of literature on mental health issues of Sri Lankan police officers revealed that, there is no published literature to explore this important occupational health issue. In the context of lack of depression related evidence base pertinent to police officers, the present study was designed to account of this scarcity by determining the prevalence of depression using the locally validated tool to assess depression. The magnitude and associations of depression elicited through the study would permit designing appropriate preventive strategies, implementing them and ultimately improving the quality of work of police officers and more importantly their well-being.

## Methods

### Study design and setting

A cross sectional study was conducted in the Kandy police division, Sri Lanka. Kandy police division is situated in the Central Province of Sri Lanka and there are twenty police stations in the Kandy police division. Kandy police division was selected for this study given its large staff, the wide range of police functions required to be carried out by the staff and the large extent of the geographic coverage. According to the Department of Census and Statistics in 2013, the mid-year population in Kandy district was 1,384,000 and the reported number of crimes was 444 per 100,000 mid-year population (http://www.statistics.gov.lk/). The study was conducted from August 2012 to January 2013, involving all twenty police stations.

### Study participants

The study population consisted of all police officers who belong to different police ranks employed in the Kandy police division at the time of the study. Altogether 2579 police officers were employed in all twenty police stations at the time of the study and these police officers were employed in different police units including protective units, crime-investigation units, law enforcement units, support units and technology infrastructure. Police officers who have been diagnosed with psychiatric disorders and police officers who were on maternity leave or on prolonged leave more than 1 month were excluded from the study. The required sample size was calculated to make estimates with a 95 % confidence level and 0.04 precision. Anticipated prevalence of depression among police officers was taken as 50 % to yield the maximum possible number, in the absence of related published literature in the South Asian context. Presuming 10 % non response rate, the sample size was computed to be 660. Considering the availability of resources and the pragmatic feasibility, a final sample of 750 police officers were recruited to the study. The sample of 750 police officers from the Kandy police division required for the study was selected using simple random sampling technique from a sampling frame prepared using the Police Officers’ register-Kandy police division.

In the final sample of 709 included for the analysis, the mean age of the police officers was 39.6 years (SD 9.2 years). The proportion of males in the sample was 83.4 % (n = 591). Ninety-eight percent (n = 697) of the police officers were Buddhists [The majority of Sri Lankans are Buddhists, which is 70.1 % according to the Census of Population and Housing 2011 (http://www.statistics.gov.lk/)]. There were 580 married police officers (81.8 %) in the sample and 85.5 % (n = 606) of police officers had at least one child.

### Study instruments

#### Associated factors

A pretested, judgemental validity assessed Self-Administered Questionnaire (SAQ) which was prepared in Sinhala, was used to collect details pertaining to socio-demographic and occupational factors from the study participants. It consisted of questions related to six basic categories, viz., socio-demographic factors, basic employment factors, work environment factors, work pattern factors, work support factors and work satisfaction factors.

#### Depression

Though depression is recognized as a major psychiatric disorder, conceptualization of depression, particularly cross-culturally, continues to be a source of discussion without unanimous consensus (Manson 1995). Numerous researches signify that cultural factors may influence the interpretation and conceptualization of their experience of depression in diverse population groups (Kleinman 2004; Chang et al. 2008; Ryder et al. 2008). In this context, in order to explore the disease or the concept of “depression”, the culturally accepted and locally validated metric tool, Peradeniya Depression Scale (PDS), was used. PDS is the first tool developed and validated in Sri Lanka in relation to depression. It is written in Sinhalese, taking into account cultural expressions and idioms of the illness and is an important, easily administrable tool to be used in research conducted in Sri Lanka. The tool was validated by comparing with the diagnosis made by consultant psychiatrists using the Structured Clinical Interview for DSM Disorders (Abeyasinghe et al. 2012). PDS consists of 25 statements to which the respondents should respond either as “yes” or “no”. At a score of 10/25 the PDS showed a sensitivity 88.5 % and specificity of 85.0 % with regard to the detection of depression (Abeyasinghe et al. 2012).

### Data analysis

Data analysis was done by using the SPSS version 16.0. After entering, double independent check of entries was carried out to identify any incompatible entries and outliers. Data related to PDS was coded (as “1” for “yes” and “0” for “no”) and entered. All the socio-demographic and occupational variables were also coded and entered.

Depression status was analyzed as a dichotomous variable. The participants who had responded with “yes” for ten or more items were categorized as depressed whereas, others were categorized as not depressed. The prevalence of depression among police officers was computed as point prevalence. The interval estimate was computed in the form of 95 % CI. As the sensitivity and specificity of PDS is known, the adjusted prevalence of depression with 95 % CI was also calculated.

Bivariate analysis was conducted using depression status as the outcome (dependent) variable and socio-demographic factors and occupational factors as the predictor (independent) variables. Depression, the outcome variable, was a dichotomous variable. Categorical data related to predictor variables were amalgamated rationally as dichotomous variables where necessary for the bivariate analysis and the crude odds ratios were calculated as the measures of effect with 95 % CI.

Given the possibility of confounding factors associated with individual predictor variables, as a means of controlling confounding, a binomial multiple logistic regression model was used. Associated factors which showed statistical significance at p < 0.10 level in the bivariate analysis were included in the multivariable analysis model using backward stepwise elimination method. The model produced adjusted odds ratios and 95 % CI with the significance level for variables of interest.

### Ethical and administrative issues

Ethical clearance to conduct the study was obtained from the Ethics Review Committee of the Rajarata University of Sri Lanka. Administrative clearance was obtained from the Inspector General of Police, Sri Lanka. Informed written consent was taken from all participants prior to data collection after explaining about the purpose and details of the study.

## Results

### Occupational characteristics of the study participants

The final sample which consisted of 709 participants represented a response rate of 94.5 %. Basic occupational characteristics of the sample of 709 police officers are summarized in Table 1.

**Table 1:** Occupational characteristics of the participants

Occupational characteristic	Number	Percentage (%)	p value*
Police rank			
Gazetted officers	87	12.3	402.2 <0.001
Non gazetted officers	622	87.7	
Police service experience			
Up to 20 years	392	55.3	7.7 0.006
>20 years	317	44.7	
Police rank experience			
Up to 10 years	435	61.4	36.1 <0.001
>10 years	274	38.6	
Total	709	100.0	

* p value for Chi squared test, Gazetted officers–Police officers who are ranked Sub Inspectors or above, Non-gazetted officers–Police officers who are ranked Police Sergeants

Of the total sample, 12.3 % (n = 87) of police officers were employed under the gazetted officer categories. The service experience of the police officers was studied in terms of their overall service in the police department and in the current rank. The mean duration of police service of the sample of police officers was 16.8 years (SD = 8.7 years). Three hundred and ninety-two police officers (55.3 %) in the sample had experience in policing for <20 years, while the majority of police officers (61.4 %, n = 435) had a service experience of 10 years or less in the current police rank.

### Prevalence of depression among police officers

According to the cut-off score of ten or more in the PDS, 162 police officers were identified as having depression. Hence, the prevalence of depression in the study sample of 709 police officers was 22.8 % (95 % CI 19.9–26.1 %).

However, given that the sensitivity and the specificity of PDS in detecting depression as 88.5 and 85 % respectively (Abeyasinghe et al. 2012), the adjusted prevalence of depression among the police officers in the Kandy police division was computed as 10.6 % (95 % CI 6.6–15.1 %; Speybroeck et al. 2013).

### Socio-demographic and occupational factors associated with depression

Bivariate analysis was conducted to elicit the association between depression and different socio-demographic characteristics such as age, sex, religion, education level, monthly income, marital status and number of children. Except for religion, none of the other factors emerged as significant predictors. Bivariate analysis revealed that, Buddhist police officers were having 0.2 times lower likelihood (OR 0.2; 95 % CI 0.1–0.7) of having depression as opposed to police officers of other religions. This association was statistically significant (p = 0.007).

Table 2 summarizes the results of the bivariate analysis of occupational factors and depression among the sample of police officers.

**Table 2:** Associations of occupational factors with depression in bivariate analysis

	Depression		No depression		Total		Odds ratio (95 % CI) p value
	n	(%)	n	%	n	%	
*Basic employment factor*							
Service experience							
≤20 years	90	(23.0)	302	(77.0)	392	(100.0)	1.1 (0.7–1.4) p = 0.938
>21 years	72	(22.7)	245	(77.3)	317	(100.0)	
Police station							
ASP I district	92	(24.6)	282	(75.4)	374	(100.0)	1.2 (0.9–1.8) p = 0.241
Other ASP districts	70	(20.9)	265	(79.1)	335	(100.0)	
Police rank							
Gazetted officers	15	(17.2)	72	(82.8)	87	(100.0)	0.7 (0.4–1.2) p = 0.186
Non-gazetted	147	(23.6)	475	(76.4)	622	(100.0)	
Police rank duration							
≤10 years	99	(22.8)	336	(77.2)	435	(100.0)	0.9 (0.7–1.4) p = 0.942
>11 years	63	(23.0)	211	(77.0)	274	(100.0)	
*Work environment factor*							
Infrastructure facilities							
Satisfactory	116	(21.1)	434	(78.9)	550	(100.0)	*0.7 (0.4*–*0.9)* *p* = *0.039*
Not satisfactory	46	(28.9)	113	(71.1)	159	(100.0)	
Welfare facilities							
Satisfactory	97	(19.1)	411	(80.9)	508	(100.0)	*0.5 (0.3*–*0.7)* *p* < *0.001*
Not satisfactory	65	(32.3)	136	(67.7)	201	(100.0)	
Staff adequacy							
Adequate	116	(21.5)	423	(78.5)	539	(100.0)	0.7 (0.5–1.1) p = 0.135
Not adequate	46	(27.1)	124	(72.9)	170	(100.0)	
*Work pattern factor*							
Working hours per week							
≤70 h	65	(20.8)	247	(79.2)	312	(100.0)	0.8 (0.6–1.2) p = 0.258
>70 h	97	(24.4)	300	(75.6)	397	(100.0)	
Consecutive shift work per week							
More frequently	118	(23.0)	394	(77.0)	512	(100.0)	1.1 (0.7–1.5) p = 0.840
Less frequently	44	(22.3)	153	(77.7)	197	(100.0)	
Emergency duties per month							
More frequently	117	(22.8)	397	(77.2)	514	(100.0)	0.9 (0.7–1.5) p = 0.929
Less frequently	45	(23.1)	150	(76.9)	195	(100.0)	
Special duties per month							
More frequently	127	(23.1)	423	(76.9)	550	(100.0)	1.1 (0.7–1.6) p = 0.775
Less frequently	35	(22.0)	124	(78.0)	159	(100.0)	
Night shifts per month							
Up to 9	102	(21.9)	363	(78.1)	465	(100.0)	0.9 (0.6–1.2) p = 0.424
≥10	60	(24.6)	184	(75.4)	244	(100.0)	
*Work support factor*							
Superior guidance							
More frequently	124	(24.0)	393	(76.0)	517	(100.0)	1.3 (0.8–1.9) p = 0.238
Less frequently	38	(19.8)	154	(80.2)	192	(100.0)	
External influence							
More frequently	112	(25.7)	324	(74.3)	436	(100.0)	*1.5 (1.1*–*2.2)* *p* = *0.023*
Less frequently	50	(18.3)	223	(81.7)	273	(100.0)	
Higher rank officer support							
Satisfactory	139	(22.4)	481	(77.6)	620	(100.0)	0.8 (0.5–1.4) p = 0.472
Not satisfactory	23	(25.8)	66	(74.2)	89	(100.0)	
Colleague support							
Satisfactory	144	(21.5)	526	(78.5)	670	(100.0)	*0.3 (0.2*–*0.6)* *p* = *0.001*
Not satisfactory	18	(46.2)	21	(53.8)	39	(100.0)	
Lower rank officer support							
Satisfactory	146	(22.3)	508	(77.7)	654	(100.0)	0.7 (0.4–1.3) p = 0.253
Not satisfactory	16	(29.1)	39	(70.9)	55	(100.0)	
Public support							
Satisfactory	134	(22.3)	466	(77.7)	600	(100.0)	0.8 (0.5–1.3) p = 0.443
Not satisfactory	28	(25.7)	81	(74.3)	109	(100.0)	
*Work satisfaction factor*							
Salary							
Satisfactory	33	(16.3)	170	(83.7)	203	(100.0)	*0.6 (0.4*–*0.9)* *p* = *0.009*
Not satisfactory	129	(25.5)	377	(74.5)	506	(100.0)	
Allowances							
Satisfactory	42	(16.7)	209	(83.3)	251	(100.0)	*0.6 (0.4*–*0.8)* *p* = *0.004*
Not satisfactory	120	(26.2)	338	(73.8)	458	(100.0)	
Social status							
Satisfactory	128	(20.3)	503	(79.7)	631	(100.0)	*0.3 (0.2*–*0.5)* *p* < *0.001*
Not satisfactory	34	(43.6)	44	(56.4)	78	(100.0)	
Public service							
Satisfactory	148	(21.6)	538	(78.4)	686	(100.0)	*0.2 (0.1*–*0.4)* *p* < *0.001*
Not satisfactory	14	(60.9)	9	(39.1)	23	(100.0)	
Overall satisfaction							
Satisfactory	125	(21.2)	465	(78.8)	590	(100.0)	*0.6 (0.4*–*0.9)* *p* = *0.020*
Not satisfactory	37	(31.1)	82	(68.9)	119	(100.0)	

Significant predictors identified in bivariate analysis at p < 0.10 are highlighted in italics

The satisfaction with the available infrastructure facilities and welfare facilities were associated with lower likelihood of having depression. In addition, having to work under external influences more frequently was found to be a statistically significant factor which has a higher likelihood to have depression. Study further revealed that, satisfactory support from colleagues was a statistically significant predictor of not having depression. Police officers who were satisfied with their salary, allowances, social status and the opportunity to serve public in relation to their jobs were less likely to have associated depression. In the study sample, police officers who had overall job satisfaction showed lower likelihood of having depression in comparison to those who were not satisfied with their job. All the above associations were statistically significant at p < 0.05 level.

These associated factors which showed statistical significance at p < 0.10 level were analyzed in the multivariable analysis and the results are summarized in Table 3. Out of ten factors, which included one socio-demographic and nine occupational factors included in the analysis, three factors were retained in the final model. Availability of satisfactory welfare facilities showed a statistically significant association in the multivariable analysis (p = 0.001). Satisfactory welfare facilities was associated with a lower likelihood (adjusted OR 0.5; 95 % CI 0.3–0.7) in comparison to unsatisfactory facilities. In relation to work satisfaction factors, police officers who were satisfied with the social status were 50 % less likely to have depression as opposed to their counterparts who were not satisfied with their social status (adjusted OR 0.5; 95 % CI 0.3–0.8). This association was statistically significant (p = 0.004). The analysis further revealed that opportunity to serve public as a significant predictor (p = 0.003) associated with lower likelihood to have depression (adjusted OR 0.2; 95 % CI 0.1–0.6).

**Table 3:** Associations of socio-demographic and occupational factors with depression in multivariable analysis

Variable	B	SE	Wald	*df*	p value	Adjusted odds ratio (95 % CI)
Work environment factor						
Welfare facilities (satisfactory)	−0.669	0.194	11.900	1	*0.001*	*0.5 (0.3*–*0.7)*
Work satisfaction factor						
Social status (satisfactory)	−0.771	0.271	8.088	1	*0.004*	*0.5 (0.3*–*0.8)*
Public service (satisfactory)	−1.388	0.471	8.669	1	*0.003*	*0.2 (0.1*–*0.6)*

Significant predictors identified in bivariate analysis at p < 0.10 are highlighted in italics

## Discussion

Though, depression has been extensively explored in a wide range of population groups across the globe, this is the first study conducted in Sri Lanka with a view to exploring depression in policing.

The prevalence (as well as the adjusted prevalence) of depression in the study sample of 709 police officers was higher than that of the findings of other studies conducted in Sri Lankan context. A cross-sectional population-based twin study with a comparable non-twin sample, conducted in Colombo District revealed that the lifetime prevalence of depression to be 6.6 % (Ball et al. 2010). A clinic based study conducted revealed 10.4 % patients were having severe depression with psychotic features at the time of presentation (Rajapakse and Sivapalasingam 2011). The diversity in the socio-demographic, cultural and economic backgrounds of the study participants over different studies would have accounted for the differences in the estimates of prevalence. The heterogeneity in the form of occupational background should be also considered as a possible explanation for the observed differences. Another important aspect which should be taken into account is the difference in assessment tools used across these studies.

In comparing with studies conducted among police officers in other countries, a study conducted in Taiwan has revealed the prevalence of major depression was 21.6 % (Chen et al. 2006). This finding is in accordance with the figures of the present study. However, a study conducted among a group of Australian police officers, the depression prevalence ranged from 37.2 to 65.5 % (Lawson et al. 2012). The differences in the nature, complexity of work and work setting among the study samples and the heterogeneity in the different assessment tools used might have accounted for these differences observed in the estimates.

In the multivariable analysis, it was found that satisfactory welfare facilities at work place was negatively associated with depression. Depression is a state which is associated with loss of interests or pleasure. As a result, the depressed workers are engaged in work with lack of concern, interest and enthusiasm. Nor do they contribute to the productivity of the organization unless they receive gratification and support in return. Hence, they tend to seek comfortable working environment which facilitates them to do their work with minimal trouble. Provision of welfare facilities at workplace is an integral aspect of promotion of well-being at the working environment. Previous studies have found that routine occupational stress or stressful work conditions have adverse effects on mental health (Liberman et al. 2002). Furthermore, a survey conducted among experienced police officers indicated that perceived work stress was significantly associated with increased depression and other related psychosomatic manifestations (Gershon et al. 2002). These findings collectively explain the present study finding.

Satisfaction with the opportunity to serve the public and satisfaction related to social status gained in policing were identified as significant occupational factors that lowered the likelihood of being categorized as having depression. The negative association of above stated factors with depression is explainable by the Conservation of Resources Model (Hobfoll 1989). This resource-oriented model is based on the supposition that people strive to retain, protect, and build resources and that what is threatening to them is the potential or actual loss of these valued resources. According to the model, individuals seek to acquire and maintain resources, such as objects, personal characteristics, conditions and energies. Provided that there is a loss of resources, or a threat of loss would result in job dissatisfaction, anxiety, and/or depression. Furthermore, studies reveal that high public demands on police officers and a mounting focus on police efficiency and integrity also contribute to the stress in this profession (Newman and Rucker-Reed 2002; Collins and Gibbs 2003). Hence, it can be postulated that when having personal satisfaction with the opportunity to serve the public promotes mental well-being.

Having used a locally validated and culturally accepted tool with high measures of diagnostic accuracy for the assessment of depression increases the internal validity of study findings. Given the fact that the study was conducted among a large group of randomly selected police officers coupled with a very high response rate enhances the generalizability of the study findings in the local context.

However, extrapolation of the findings across nations has limitations owing to the heterogeneity of factors associated with depression across large geographic areas. Albeit this limitation, the findings could be applicable in countries which have similar policing work profiles to that of Sri Lanka. Thus, the findings could be having practical implications in exploring the predictors of depression among law enforcing officers working in South Asian countries which have similar job demands, work profiles and work stressors. Furthermore, given the evident similarities in the contexts of social impression and media criticism associated with policing in these countries, the findings of the present study could be applicable for other countries in the region.

Due to the cross sectional nature of the study, temporal relationship between depression and associated factors cannot be commented based on the present study. This study was limited only to some selected socio-demographic factors and occupational factors. Some of the personal health factors, behavioural factors, personal life factors and coping strategies that could have been elicited as possible predictors of depression were not considered in this study. While being predictors of depression, they could also have been confounders and effect modifiers. Their effects could not be taken into account when the associations were adjusted for confounding. This has to be taken into account when interpreting results. Though confounding was accounted for by multivariable analysis, the effect of unknown confounders could not be accounted for. Additionally, this analysis did not consider interactions and additive effects between variables.

## Conclusions

The prevalence of depression among police officers in the Kandy police division was found to be high in comparison to the reported rates in other studies in Sri Lankan context. This signifies that the magnitude and the associations of depression in policing should be further explored with a holistic approach to uplift the overall well-being of the target population. Provided that some occupational factors identified as predisposing factors at the level of bivariate analysis for depression are modifiable, it is recommended to design a package of interventions and implement adaptive measures to rectify the problems related to depression among police officers.

It is recommended to design and implement further observational research, in order to identify different facets of associated factors in relation to depression. Longitudinal research is preferable with the view to identifying the temporal associations of these predictors.

## Appendix: The PDS translated into English

The following questions are about you have been feeling over the past 2 weeks. Please consider how you have felt over the past 2 weeks, and circle the most appropriate response for each statement.

1. Have you been experiencing burning pains of your body? Yes/No
2. Have you been having difficulty sleeping at night? Yes/No
3. Have you lost weight, without an obvious illness? Yes/No
4. Have you been experiencing loss of appetite? Yes/No
  Have you often been experiencing any of the following?
5. Headache Yes/No
6. Chest ache or palpitations Yes/No
7. Aches of the arms or legs Yes/No
8. Aches of the body Yes/No
9. Fullness of the abdomen Yes/No
10. Faintishness Yes/No
  Have you been suffering from any of the following?
11. Grief-sadness Yes/No
12. Distress-low mood Yes/No
13. Increased anger or irritability Yes/No
14. Sadness Yes/No
15. Have you been feeling sad that there is no point in living anymore? Yes/No
16. Have you been fearful about the future of yourself or your family? Yes/No
17. Have you felt uncertain, or felt less confident in yourself? Yes/No
18. Have you felt suspicious at times? Yes/No
  Have you been worrying that the above difficulties are due to any of the following?
19. Due to the affect of a previous karma Yes/No
20. Because of an astrologically bad period Yes/No
21. Because of evil charms cast against you Yes/No
22. Have you been unable to attend to your usual everyday work at home? Yes/No
23. Have you been unable to do your job as usual? Yes/No
24. Have you found yourself avoiding the company of other people? Yes/No
25. For some time now, have you been seeking treatment for an illness that could not be identified? Yes/No

## Abbreviations

95 % CI: 95 % confidence interval
OR: odds ratio
PDS: Peradeniya Depression Scale
SAQ: Self-Administered Questionnaire
